# Regulation of NOX/p38 MAPK/PPARα pathways and miR-155 expression by boswellic acids reduces hepatic injury in experimentally-induced alcoholic liver disease mouse model: novel mechanistic insight

**DOI:** 10.1007/s12272-023-01441-6

**Published:** 2023-03-23

**Authors:** Rania M. Salama, Samah S. Abbas, Samar F. Darwish, Al Aliaa Sallam, Noura F. Elmongy, Sara A. El Wakeel

**Affiliations:** 1 Pharmacology and Toxicology Department, Faculty of Pharmacy, Misr International University (MIU), KM 28, Cairo-Ismailia Road, Ahmed Orabi District, Cairo, Egypt; 2 Pharmacology and Toxicology Department, Faculty of Pharmacy, Badr University in Cairo (BUC), Cairo, Egypt; 3 Biochemistry Department, Faculty of Pharmacy, Ain Shams University, Cairo, Egypt; 4 Physiology Department, Damietta Faculty of Medicine, Al-Azhar University, Damietta, Egypt

**Keywords:** Boswellic acids, Alcoholic liver disease, NOX, PPARα, miR-155

## Abstract

Alcoholic liver disease (ALD) refers to hepatic ailments induced by excessive alcohol intake. The pathogenesis of ALD comprises a complex interplay between various mechanistic pathways, among which inflammation and oxidative stress are key players. Boswellic acids (BAs), found in *Boswellia serrata*, have shown hepatoprotective effects owing to their antioxidant and anti-inflammatory activities, nevertheless, their therapeutic potential against ALD has not been previously investigated. Hence, this study was performed to depict the possible protective effect of BAs and detect their underlying mechanism of action in an experimentally-induced ALD mouse model. Male BALB/c mice were equally categorized into six groups: control, BAs-treated, ALD, and ALD that received BAs at three-dose levels (125, 250, and 500 mg/kg) by oral gavage for 14 days. Results showed that the high dose of BAs had the most protective impact against ALD according to histopathology examination, blood alcohol concentration (BAC), and liver function enzymes. Mechanistic investigations revealed that BAs (500 mg/kg) caused a significant decrease in cytochrome P450 2E1(CYP2E1), nicotine adenine dinucleotide phosphate oxidase (NOX) 1/2/4, p38 mitogen-activated protein kinase (MAPK), and sterol regulatory element-binding protein-1c (SREBP-1c) levels, and the expression of miR-155, yet increased peroxisome proliferator-activated receptor alpha (PPARα) levels. This led to an improvement in lipid profile and reduced hepatic inflammation, oxidative stress, and apoptosis indices. In summary, our study concludes that BAs can protect against ethanol-induced hepatic injury, via modulating NOX/p38 MAPK/PPARα pathways and miR-155 expression.

**Supplementary Information:**

The online version contains supplementary material available at 10.1007/s12272-023-01441-6.

## Introduction

It is estimated that 2.4 billion people throughout the globe drink alcoholic beverages (Asrani et al. 2021). In addition, over 75 million suffer from alcohol intake problems and are liable to alcohol-induced liver diseases. Around 90–98% of total alcohol consumption undergoes metabolism in the liver. Accordingly, excessive alcohol consumption can lead to obvious hepatic damage (Kawaratani et al. 2017).

Alcoholic liver disease (ALD) is an umbrella term that encompasses a broad range of hepatic injuries, ranging from steatosis to a fatal hepatocellular carcinoma (Namachivayam and Valsala Gopalakrishnan 2021). The pathogenesis of ALD is a complex interplay between inflammation and oxidative stress, that involves direct alcohol-induced hepatotoxicity, ensued by the elevation of reactive oxygen species (ROS), a decline in the antioxidant capacity, and buildup of fats in liver cells, in addition to the Kupffer cells (KCs)-mediated inflammation (Namachivayam and Valsala Gopalakrishnan 2021). Ethanol is metabolized by alcohol dehydrogenase (ADH) and the liver microsomal cytochrome P450 2E1 (CYP2E1) yielding acetaldehyde and ROS. The nicotine adenine dinucleotide phosphate oxidase (NOX) enzyme system is one of the key sources of ROS in hepatic stellate cells (HSCs) and KCs.

Ethanol intake triggers KCs-induced inflammation, as NOX-derived ROS play a prominent role in activating nuclear factor kappa B (NFκB) and tumor necrosis factor-alpha (TNF-α) in KCs (Yang et al. 2022). ROS elevation will stimulate apoptotic pathways as revealed through the increased caspase-3 activity in the liver cells, in response to enhanced p38 mitogen-activated protein kinase (MAPK). Likewise, alcoholic consumption elicits hepatic fat accumulation through an elevation in sterol regulatory element-binding protein-1c (SREBP-1c), inducing the expression of lipogenesis genes, and halting the expression of genes taking part in β-oxidation of fatty acid such as peroxisome proliferator activator receptor alpha (PPARα) target genes (Yang et al. 2022). Furthermore, chronic alcohol consumption elicits lipid peroxidation, thus, increasing malondialdehyde (MDA) and 4-hydroxynonenal (4-HNE) (Li et al. 2019).

Micro RNA (miR) plays an obvious role in the derangement of liver metabolism and liver injury via the regulation of gene expression (Wang et al. 2021b). MiR-155 is one of the markedly upregulated miRNAs in rats with fatty liver. It was also revealed that miR-155-knockout mice with fatty liver showed a decline in hepatic steatosis in addition to a reduced expression of the proteins implicated in fatty acid metabolism (Zhang et al. 2020).

The availability of specific medications that can halt the progression of ALD is currently missing, which makes abstinence from alcohol as the most efficient and safe regimen for ALD. This instigates the introduction of novel strategies to combat ALD employing the antioxidant, anti-inflammatory, and anti-apoptotic potential of various herbal remedies and making use of their high safety margin (Gao and Bataller 2011; Ming et al. 2021).

The gum resin extract of *Boswellia serrata* (*B. serrata*), rich in boswellic acids (BAs), is known for its protective and therapeutic benefit in multiple inflammatory and non-inflammatory diseases including diabetes, asthma, cancer, arthritis, and inflammatory bowel disease (Roy et al. 2019; Zhang et al. 2019). Terpenoids, phenolic compounds, flavonoids, and phenylpropanoids, the main components present in *B. serrata*, were shown to be responsible for its beneficial effects (Ayub et al. 2018). Importantly, some BAs have gained considerable notice, and previous reports have highlighted the protective impact of BAs on diet-induced non-alcoholic liver disease (Zaitone et al. 2015; Katragunta et al. 2019). Yet, its impact on experimentally-induced ALD has not been previously investigated. Therefore, in the present study, NOX/p38 MAPK/PPARα stream is investigated as one of the tracks that may define the hepatoprotective effect of BAs in a mouse model of ethanol-induced liver injury.

## Materials and methods

### Ethics statement

The study was performed according to the ARRIVE guidelines (Kilkenny et al. 2010), and all procedures were approved by the institutional ethics committee for the care and use of animals of Damietta Faculty of Medicine, Al-Azhar University, Egypt (Approval no.: IRB 00012367-22-01-001). All efforts were made to minimize animal suffering and to decrease the number of animals used.

### Animals

Forty-eight adult (≈ 8–10 week-old) male BALB/c albino mice (22 ± 2 g) were purchased from The Nile Company for Pharmaceuticals and Chemical Industries (Cairo, Egypt). Mice were housed in standard polypropylene cages and were acclimatized for one week before the start of the experiment. Mice were allowed free access to a normal pellet diet (EL Nasr Pharmaceutical Chemicals Co., Cairo, Egypt) and tap water throughout the experimental period. The mice were kept under standard controlled temperature (22 ± 2 °C) and relative humidity (55 ± 5%) at a 12-light/12-dark cycle.

### Drugs and chemicals

*Boswellia serrata*-standardized extract containing 65% BAs was obtained from Swanson® Health Products (Fargo, ND, USA) in the form of capsules. The powder was evacuated from the capsules, then suspended in distilled water to reach a final concentration of 50 mg/ml.

Ethanol 95% was purchased from Sigma-Aldrich, St. Louis, MO, USA, while maltose-dextrin (Cat. # 3653) was purchased from Bio-Serv (Flemington, NJ, USA).

### Induction of alcoholic liver disease (ALD)

Chronic alcohol plus binge feeding was used to induce ALD, based on The National Institute on Alcohol Abuse and Alcoholism (NIAAA) Model (Guo et al. 2018) that utilized Lieber-DeCarli liquid diet. The ALD group was fed a freshly prepared ethanol liquid diet everyday (Cat. # F1258SP), purchased from Bio-Serv, Flemington, NJ, USA. Mice were gradually acclimatized to the liquid diet by introducing an alcohol-free diet on day one, then an increasing volume of 95% ethanol from 1 to 4% (v/v) from day two to five, and finally 5% (v/v) ethanol from day six to fifteen. On day sixteen, mice received a single binge 31.5% (v/v) alcohol dose (5 g/kg; p.o.) by oral gavage, which is equivalent to 6.33 ml/kg.

The control groups received a control liquid diet (Cat. # F1259SP) freshly prepared daily, purchased from Bio-Serv, Flemington, NJ, USA, for the same duration. The control liquid diet formula included maltose-dextrin instead of ethanol in the same isocaloric amounts, and on the last day, control mice received a single oral gavage of the same volume of maltose-dextrin.

To ensure an equal intake of the liquid diet, mice were housed individually, and each mouse was fed a 20–25 ml of diet using the graduated Bio-Serv™ Liquid Diet Feeding Tubes (NJ, USA). Also, any remaining volume from the previous day is added to the volume of the next daily diet.

### Experimental design

The study was carried out in two phases:

#### Phase A- dose-response screening study

Mice were randomly divided into 6 groups (n = 8) and received the liquid diet and treatments for 14 days as follows:

##### Group 1

Control mice were fed the control liquid diet through the feeding tubes and received distilled water (10 ml/kg/day; p.o.) by oral gavage.

##### Group 2

Mice were fed the control liquid diet using the feeding tubes and received BAs (500 mg/kg/day; p.o.) by oral gavage.

##### Group 3

Mice were fed the ethanol-containing liquid diet using the feeding tubes to induce experimental ALD.

##### Group 4, 5, and 6

Mice were fed the ethanol-containing liquid diet using the feeding tubes and received BAs (125, 250, and 500 mg/kg/day; p.o.) by oral gavage. Dose selection of BAs was based on previous studies reporting the protective effects of BAs (Barakat et al. 2018; Sami et al. 2019).

On day 15, a single dose of binge ethanol was given by oral gavage to groups 3–6, while control groups (groups 1 and 2) received the same volume of maltose-dextrin. One hour later, all mice were weighed, and blood was withdrawn using heparinized capillary tubes from the retro-orbital plexus after anesthesia with ketamine/xylazine (100 mg/10 mg; i.p.) cocktail (Hector et al. 2020) to separate sera. Lastly, all animals were euthanized by cervical dislocation, and livers were rapidly dissected, dried between two filter papers, and weighed for the assessment of liver index. Subsequently, livers were divided into 2 subsets: the first one comprised one lobe of each mouse liver that was immersed in 10% neutral buffered formalin to be reserved for histopathology and immunohistochemistry examination. The second subset comprised the other two lobes of livers of all mice, which were flash-frozen in liquid nitrogen and stored at − 80 °C for later biochemical assessments.

Dose-response effect of BAs was determined according to serum levels of liver function enzymes, blood alcohol concentration (BAC), and histopathological examination.

#### Phase B- investigating the hepatoprotective mechanism of boswellic acids in ethanol-induced alcoholic liver disease in mice

Based on the results of the preliminary dose-response study, the most hepatoprotective dose was the high one (500 mg/kg), thus was selected for further investigations to assess its hepatoprotective mechanism.

## Methods

### Change in body weight and liver index

Body weight change was calculated as the difference between the initial and final body weight before sacrifice.

The liver index was calculated according to the following formula:

Liver index = [Liver weight (g)/final body weight (g)] × 100 (Qu et al. 2019).

### Assessment of liver function enzymes

Serum concentrations of alanine aminotransferase (ALT) and aspartate aminotransferase (AST) were determined by colorimetric assay using available commercial kits (Teco Diagnostics, Anaheim, CA, USA).

### Assessment of blood alcohol concentration (BAC)

Blood alcohol concentration (BAC) was assessed in serum by an ultrasensitive colorimetric EnzyChrom™ ethanol assay Kit (Bioassay Systems, Hayward, CA, USA).

### Assessment of lipid profile

Serum concentrations of total cholesterol (TC), high-density lipoprotein-cholesterol (HDL-C), and low-density lipoprotein-cholesterol (LDL-C) were determined by colorimetric assay using available commercial kits supplied from BioChain, Hayward, CA, USA. Meanwhile, triglycerides (TG) were determined by an enzymatic assay kit supplied by XpressBio Life Science Products, Thurmont, MD, USA.

### Assessment of hepatic alcohol metabolizing enzyme activities

The hepatic alcohol dehydrogenase (ADH) and aldehyde dehydrogenase (ALDH) activities were assessed as previously described by (Li et al. 2021).The assay of ADH activity depends on observing the conversion of NAD^+^ to NADH by recording the changes in the absorbance at 340 nm for 5 min after the initiation of the enzyme reaction. As for the ALDH activity, the assay depends on observing the conversion of acetaldehyde and NAD^+^, catalyzed by ALDH, into acetic acid and NADH by recording the changes in the absorbance at 340 nm for 5 min after the initiation of the enzyme reaction.

### Assessment of oxidative stress markers in hepatic tissue

Hydrogen peroxide (H_2_O_2_) levels were measured in hepatic tissues using a colorimetric assay kit (Abcam, Cambridge, UK, Cat. # ab102500). The colored product was measured at 470 nm.

Sandwich enzyme-linked immunosorbent (ELISA) assay was used for assessment of hepatic catalase (CAT) activity (LifeSpan Biosciences, Seattle, WA, USA, Cat. # LS-F34515), superoxide dismutase (SOD) activity (MyBioSource, San Diego, CA, USA, Cat. # MBS034842), and glutathione peroxidase (GPx) activity (MyBioSource, San Diego, CA, USA, Cat. # MBS700004). Moreover, a competitive ELISA technique using kits from MyBioSource (San Diego, CA, USA) was used for the measurement of MDA (Cat. # MBS741034) and 4-HNE levels (Cat. # MBS7606509). All ELISA procedures were performed according to the manufacturer’s directions.

### Enzyme-linked immunosorbent assay (ELISA) for TNF-α, IL-1β, BAX, BCL2, CYP2E1, adiponectin, and CXCL1

Assessment of the inflammatory markers, TNF-α and IL-1β, in liver homogenate was done by sandwich ELISA assay kits supplied by MyBioSource, San Diego, CA, USA, Cat. # MBS825075, and by Cusabio, Houston, TX, USA, Cat. # CSB-E08054m, respectively.

For the apoptotic markers, determination of BCL2-associated X protein (BAX) and B-cell lymphoma 2 (BCL2) was carried out by sandwich ELISA method using kits obtained from MyBioSource, San Diego, CA, USA, Cat. # MBS2509733, and from EIAab Science, Wuhan, China, Cat. # E0778m; correspondingly.

Hepatic tissue CYP2E1, adiponectin, and C-X-C motif chemokine ligand 1 (CXCL1) were analyzed by sandwich ELISA kits bought from MyBioSource, San Diego, CA, USA, Cat. # MBS453581; LifeSpan Biosciences, Seattle, WA, USA, Cat. # LS-F2600; and R&D systems, Minneapolis, MN, USA, Cat. # MKC00B, respectively.

### Quantitative real-time reverse-transcription PCR assessment of miRNA-155 expression in hepatic tissue

Total RNA isolation (TRIzol) reagent (Invitrogen GmbH, Darmstadt, Germany, Cat. # 15596-026) was used to isolate total RNA from each hepatic tissue sample. The isolated RNA pellets were treated with an RNase-free DNase kit (Qiagen, Hilden, Germany). The RNA concentrations (ng/mL) and purities of all aliquots were determined using NanoDrop 1000 spectrophotometer (Thermo Scientific, Wilmington, DE, USA). The isolated RNA aliquots were kept at 80^°^C till the reverse transcription step. Complementary DNA (cDNA) was synthesized from isolated RNA using the RevertAid First Strand cDNA Synthesis Kit (Cat. # K1622, Thermo Fisher Scientific Inc., Waltham, MA, USA) according to the manufacturer’s directions. Afterward, the reaction tubes containing cDNA were collected on ice for cDNA amplification. Amplifications were performed by using miScript SYBR Green PCR Kit and TaqMan™ MicroRNA Assay (Thermo Fisher Scientific Inc., Waltham, MA, USA), based on the manufacturer’s guidelines. RNU6B (U6) was used as an internal control for data normalization. The mature qRT-PCR primer sequence of miR-155 (GenBank Accession no.: **NR_029565**) was: 5′- UUAAUGCUAAUUGUGAUAGGGGU-3′, while the primer sequence of acetyl-CoA carboxylase-1 (ACC-1) (GenBank Accession no.: **AY451393.1**) was: forward: GGAGATGTACGCTGACCGAG, reverse: TCACTGCGCCTTCAACTTCT, fatty acid synthase (FASN) (GenBank Accession no.: **NM_007988.3**) was: GGGTGTGCCATTCTGTCAGT, reverse: GGCCTTGTGACAGTCTCTCC, and U6 (GenBank Accession no.: **K00784.1**) was: forward: CTCGCTTCGGCAGCACA, reverse: AACGCTTCACGAATTTGCGT.

The relative expression of miR-155 to U6 was calculated using the Eq. 2^−ΔCt^, where ΔCt = (CtmiR-155- CtU6). The fold change of miR-155 was determined by the 2^−ΔΔCt^ method as previously described (Livak and Schmittgen 2001).

### Western blot analysis

The ReadyPrep™ total protein extraction kit, provided by Bio-Rad Laboratories, Inc., Hercules, CA, USA (Cat. # 163–2086), was employed to extract protein from tissue homogenate according to the manufacturer’s instructions. Bradford Protein Assay Kit (SK3041) for quantitative protein analysis was provided by Bio Basic Inc. (Markham, Ontario L3R 8T4, Canada). Then, protein samples were loaded (100 µg/well) on 10% SDS-PAGE gel. Loading of samples was done on two separate gels and separated simultaneously using a Mini-PROTEAN Tetra Cell (Bio-Rad Laboratories, Inc., Hercules, CA, USA). After electrophoresis, each gel was transferred onto a corresponding activated polyvinylidene difluoride (PVDF) membrane (Cat. # ab133411, Abcam, Cambridge, UK). Membranes’ blocking was then done by tris-buffered saline with Tween 20 (TBST) and 3% bovine serum albumin (BSA). The first membrane was then cut exactly at molecular weights (75 and 55 kDa) into 3 pieces. The first piece > 75 kDa was incubated overnight with the primary rabbit polyclonal antibody against SREBP-1c at 1: 1000 (Cat. # ab28481), the second piece > 55 kDa incubated with antibody against NOX1 at 1:500 (Cat. # ab131088), and the third piece < 55 kDa incubated with antibody against p-p38 MAPK (T180/Y182) at 1:000 (Cat. # ab45381) and p38 MAPK at 1:500 (Cat. # ab170099). After washing, the membrane pieces were incubated with goat anti-rabbit HRP-conjugated secondary antibody (Cat. # ab6721) at 1:5000. The chemiluminescent substrate (Clarity™ Western ECL substrate, Bio-Rad, Laboratories, Inc., Hercules, CA, USA, Cat. # 170–5060) was applied to the blot according to the manufacturer’s recommendations. Afterward, the second and third membrane pieces were stripped and re-probed (> 55 kDa) with rabbit polyclonal NOX2 antibody at 1:500 (Cat. # ab129068), while the other piece (< 55 kDa) was probed with anti-β-actin antibody (Cat. # ab8227), as a loading control. The second PVDF membrane was incubated with rabbit polyclonal NOX4 (Cat. # ab154244). After developing, it was stripped and re-probed against rabbit polyclonal PPARα (Cat # ab227074). All antibodies were obtained from Abcam, Cambridge, UK.

### Determination of protein content

Assessment of protein content in mice livers was done using a protein assay kit according to the manufacturer’s instructions (Cat. # 23227), purchased from Thermo Fisher Scientific (Waltham, MA, USA). Bicinchoninic acidbased protein assay was used for the recognition and quantitation of total protein colorimetrically (Gornall et al. 1949).

### Histopathological examination

Liver tissue samples were dissected and fixed in 10% neutral buffered formalin for 72 hrs. Then, the samples were processed and dehydrated in serial grades of ethanol, cleared in xylene, impregnated, and embedded in Paraplast tissue embedding media. Five µm-thick tissue sections were cut by rotatory microtome and mounted on glass slides, then stained by Hematoxylin and Eosin (H&E) as a general histological examination staining method. All standard procedures for sample fixation and staining were according to Culling (2013).

### Immunohistochemical detection of phospho-nuclear factor kappa B p65 (Ser276) and cleaved caspase-3

According to the manufacturer’s protocol, deparaffinized five µm-thick tissue sections were treated with 3% H_2_O_2_ for 20 min., washed, and then incubated with anti-p-NFκB p65 (Ser276) (GeneTex Inc., Irvine, CA, USA) (Cat. # GTX54672, 1:100) and anti-cleaved caspase-3 antibody (Cell Signaling Technology, Danvers, MA, USA) (Cat. # Asp175, 1: 200) overnight at 4 °C. Afterward, tissue sections were washed with PBS, incubated with secondary antibody horseradish peroxidase (HRP) EnVision kit (Dako, Glostrup, Denmark) for 20 min., then washed with PBS and incubated with 3,3′-diaminobenzidine (DAB) for 10 min., then washed again with PBS and counter-stained with hematoxylin, dehydrated, and cleared in xylene, and finally, cover-slipped for microscopic examination.

According to the method Elsayed et al. (2022), eight representative non-overlapping fields were randomly selected and scanned per tissue section of each sample for the determination of the area-based percentage of immunohistochemical expression levels of p-NFκB p65 and cleaved caspase-3 in immune-stained sections. Data were obtained using a full HD microscopic imaging system (Leica Microsystems GmbH, Wetzlar, Germany) operated by Leica application software for tissue section analysis.

### Statistical analysis

The Kolmogorov-Smirnov test was employed to test each variable for normality. As all data were normally distributed, parametric analysis was performed. Data were expressed as means ± SD and compared using one-way ANOVA followed by Tukey’s *post hoc* test. Differences were considered statistically significant at a level of probability (*p*-value) less than 0.05. Statistical analysis was performed using the statistical software package GraphPad Prism^®^, version 8.00 for Windows (California, USA).

## Results

The statistical comparison between the control and BAs (500 mg/kg/day) groups showed no significant difference, hence all comparisons were referred to the control group.

### Hepatoprotective effects of different doses of boswellic acids in ethanol-treated mice

#### Effect on body weight, liver index, alcohol concentration, and liver enzymes

The screening study unveiled that ethanol feeding led to a significant decrease in the body weight of the mice when compared to the control group. Only treatment with the high dose of BAs (500 mg/kg) achieved a significant increase in body weight when compared to the ethanol-fed mice. However, no significant change was observed in the weights of the mice of the BAs low- and medium-dose treated groups, when compared to the ethanol-fed one. Results of the liver index showed that ethanol feeding induced ≈ a 2-fold increase in the liver index when compared to the control group. Treatment with the high dose of BAs revealed a significantly lower liver index (≈ 37% decrease) when compared to both the ALD and the low-dose (ALD + BAs 125) treated groups. Yet, the low and medium doses of BAs (125 and 250 mg/kg) failed to show any significant change in the liver index, compared to the ethanol-fed group (Table 1).

**Table 1:** Dose-response effect of boswellic acids (BAs) (125, 250, and 500 mg/kg/day) on the body weight change, liver index, liver function enzymes, blood alcohol concentration (BAC), and hepatic alcohol metabolizing enzyme activities in experimentally-induced alcoholic liver disease (ALD) in mice

	Control	BAs 500	ALD	ALD + BAs 125	ALD + BAs 250	ALD + BAs 500
Body weight change (g)	3.63 ± 2.67	3.50 ± 3.07	− 5.56 ± 7.80^*^	− 3.00 ± 2.78	0.00 ± 3.11	1.38 ± 3.02^#^
Liver index (%)	5.52 ± 1.25	4.55 ± 1.05	12.26 ± 3.12^*^	12.39 ± 1.82	9.71 ± 1.62	7.77 ± 2.18^#, @^
ALT (U/L)	35.21 ± 10.25	38.27 ± 4.80	204.70 ± 28.40^*^	148.10 ± 16.10^#^	87.54 ± 11.25^#, @^	54.46 ± 12.13^#, @, $^
AST (U/L)	41.19 ± 4.65	46.66 ± 3.28	275.5 ± 24.01^*^	172 ± 12.85^#^	118.5 ± 15.03^#, @^	78.95 ± 17.51^#, @, $^
BAC (mg/dl)	77.50 ± 10.76	66.38 ± 12.25	414.40 ± 49.86^*^	340.80 ± 33.44^#^	298 ± 34.71^#^	223.90 ± 29.26^#, @, $^
ADH (nmol/mg protein/min)	4.06 ± 0.24	3.99 ± 0.22	1.31 ± 0.32^*^	1.98 ± 0.46^#^	2.15 ± 0.35^#^	2.68 ± 0.59^#, @^
ALDH nmol/mg protein/min)	4.51 ± 0.62	4.73 ± 0.68	2.07 ± 0.63^*^	2.70 ± 0.49	2.99 ± 0.63^#^	3.91 ± 0.56^#, @, $^

Data are presented as the mean ± SD (n = 8 per group; one-way ANOVA followed by Tukey’s multiple comparison test

*ADH* alcohol dehydrogenase, *ALDH* aldehyde dehydrogenase, *ALT* alanine aminotransferase, *AST* aspartate aminotransferase

^*^p < 0.05, vs. the control group; ^#^*p* < 0.05, vs. the ALD group; ^@^*p* < 0.05 vs. BAs 125 mg/kg; ^$^*p* < 0.05, vs. BAs 250 mg/kg).

Alternatively, the screening study proved that the ethanol-fed mice showed a marked increase (≈ 6, 7, and 5 folds) in serum liver function enzymes (ALT and AST) and BAC, respectively, when compared to the control mice. Administration of the three doses of BAs (125, 250, or 500 mg/kg/day) caused a significant reduction in ALT and AST serum levels with the highest effect shown in the BAs (500 mg/kg/day) group with ≈ 73% and 71% reduction, respectively, as compared to the ALD group. Similarly, the greatest effect in reducing BAC (≈ 46%) was observed in the BAs (500 mg/kg/day) group, as compared to the ALD group.

On the other hand, ethanol feeding caused a substantial fall in the hepatic alcohol metabolizing enzyme activities: ADH and ALDH by 67.7 and 54%, respectively, relative to the control group. These activities were augmented significantly upon administration of the highest dose of BAs (500 mg/kg/day) by ≈ 2-fold increase when compared to the ALD group.

#### Histopathological results of hepatic tissues

**Fig. 1:**
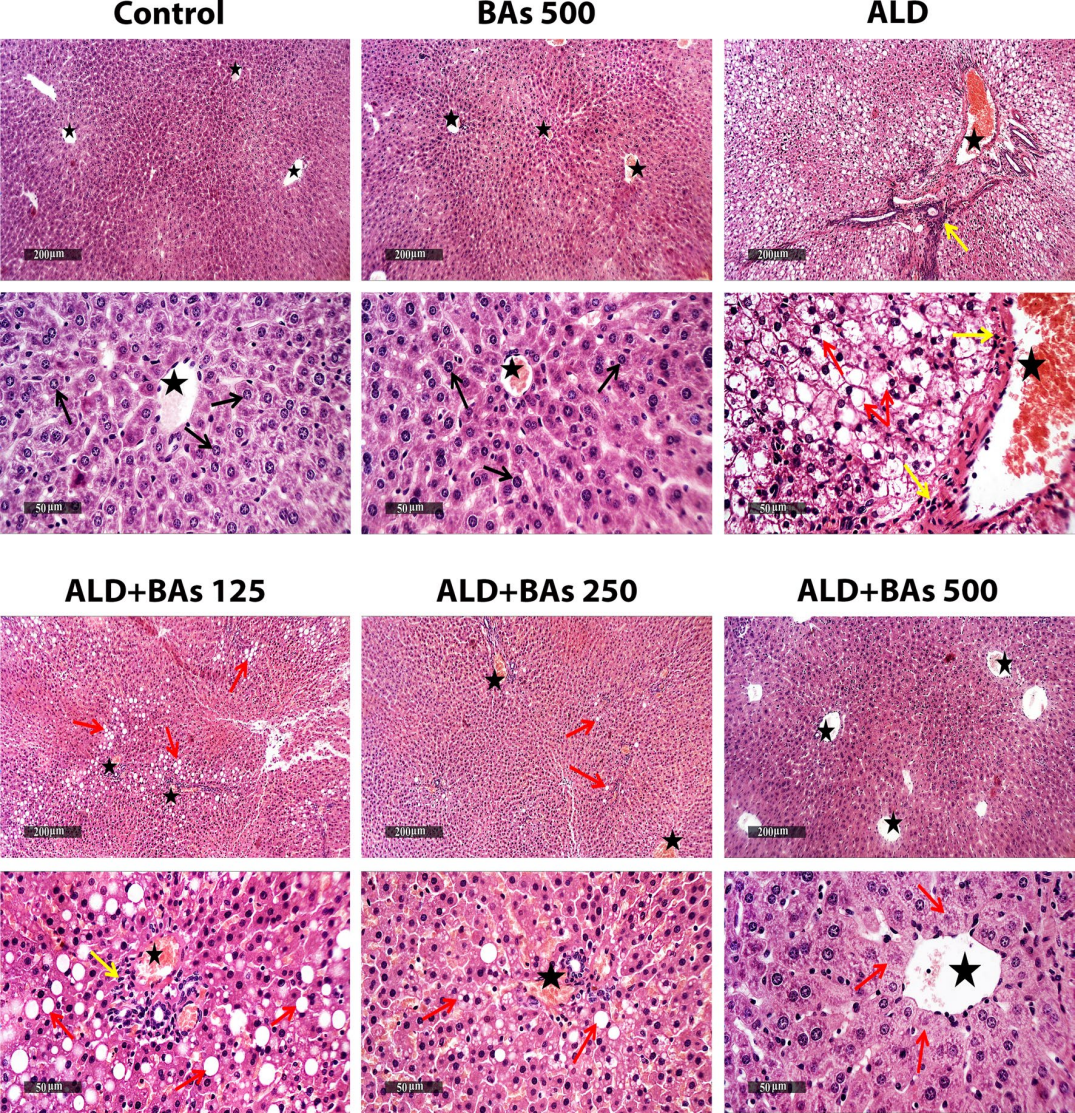
Photomicrographs of H&E-stained hepatic sections (X100 and X400) showing the effect of BAs treatment (125, 250, and 500 mg/kg) on the ethanol-induced ALD in mice. Both control and BAs-treated groups show intact hepatocytes (arrow) and vasculature (star). The ALD group shows broad areas of hepatic steatosis with many pyknotic nuclei (red arrow), moderate to severe dilatation of hepatic vasculatures (star), and inflammatory cell aggregates (yellow arrow). ALD+BAs 125 group shows moderate persistent hepatocellular macrovesicular steatosis (red arrow), congested hepatic vasculatures (star), and mild periportal inflammatory cell aggregates (yellow arrow). ALD+BAs 250 group shows occasional records of fatty changes in some hepatic lobules (red arrow) and mild persistent congested blood vessels (star). As for the ALD+BAs 500 group, almost intact morphological structures are observed, with few degenerated hepatocytes (red arrow) and intact vasculature (star). ALD, alcoholic liver disease; BAs, boswellic acids; KCs, Kupffer cells.

As illustrated in Fig. 1, microscopic examination of mice liver samples from different groups revealed that the control liver samples demonstrated normal morphological structures of mice liver parenchyma with many apparent intact well-organized hepatocytes and subcellular details, minimal degenerative changes records, intact hepatic vasculatures as well as hepatic sinusoids. Similarly, BAs (500 mg/kg/day) liver tissues showed apparent intact morphological features of hepatic parenchyma.

However, the ALD group liver samples exhibited wide areas of hepatocellular macrovesicular steatosis with many figures of pyknotic nuclei all over different zones of hepatic lobules. Moderate to severe dilatation of hepatic vasculatures were also shown, accompanied by periportal inflammatory cell aggregates.

Liver samples of the low dose (BAs 125 mg/kg) treated group showed moderate persistent figures of hepatocellular macrovesicular steatosis and pyknosis mostly at periportal zones of hepatic lobules. Moderate congested hepatic vasculatures including sinusoids were shown, accompanied by mild periportal inflammatory cell aggregates. As for the moderate dose (BAs 250 mg/kg) treated group, liver samples showed mild occasional records of fatty changes in some hepatic lobules, with mild persistent congested blood vessels and minimal inflammatory cell infiltrates. Finally, treatment with the high dose of BAs (500 mg/kg) exhibited almost intact well protected morphological features of hepatic parenchyma all over hepatic lobules and intact vasculatures with occasional records of pericentral degenerative changes of few hepatocytes.

### Assessment of the mechanisms underlying boswellic acids’ protective effects against ethanol-induced alcoholic liver disease in mice

#### Effect of boswellic acids on lipid profile (TC, TG, HDL-C, and LDL-C) of ethanol-treated mice

**Fig. 2:**
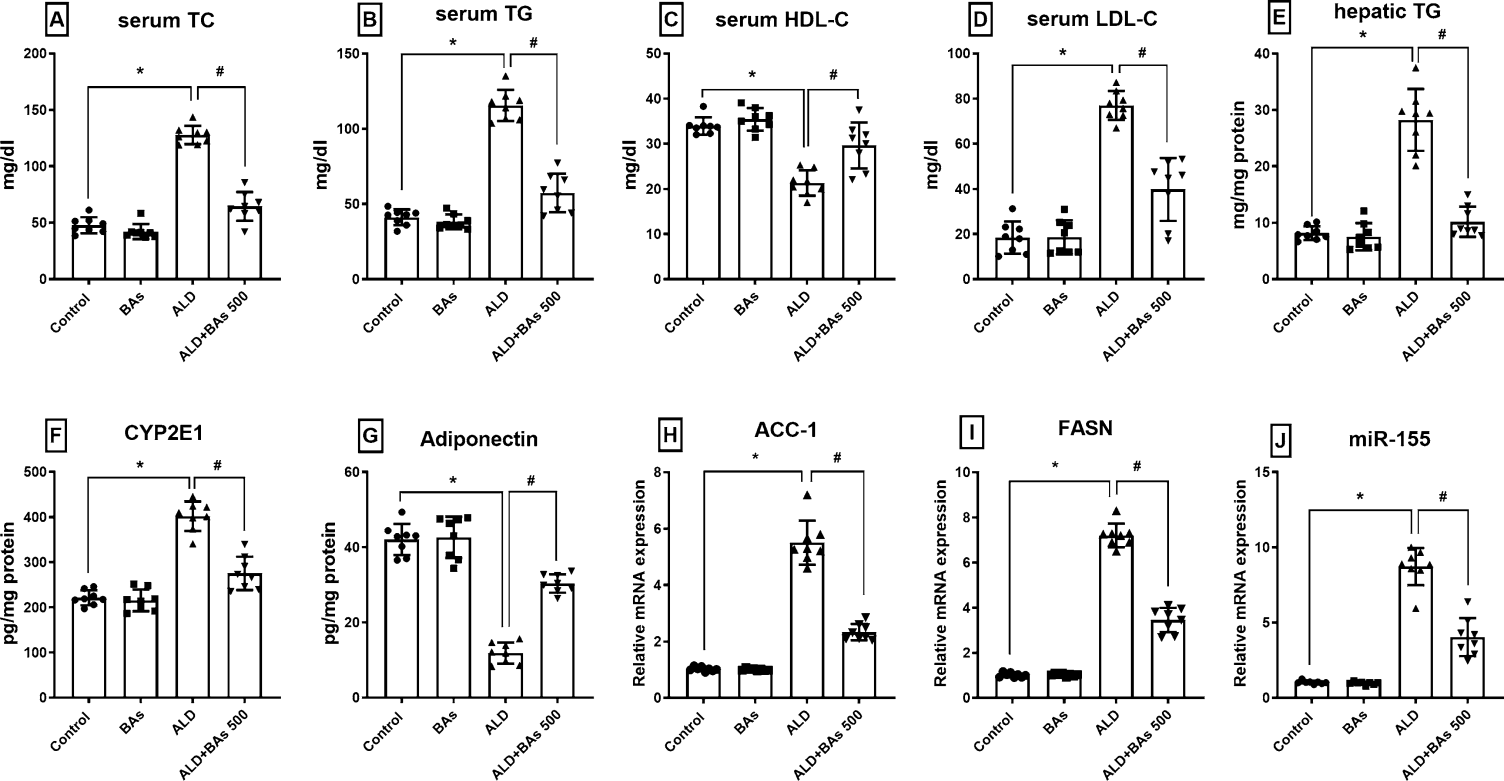
Effect of BAs (500 mg/kg) on the serum levels of **A** TC, **B** TG, **C** HDL-C, **D** LDL-C, and hepatic levels of **E** TG, **F** CYP2E1, **G** adiponectin, and relative gene expression of **H** ACC-1, **I**, FASN and **J** miR-155 in experimentally-induced ALD in mice. Data are presented as the mean ± SD (n = 8 per group; one-way ANOVA followed by Tukey’s multiple comparison test; ^*^p < 0.05, vs. the control group; and ^#^*p* < 0.05, vs. the ALD group). ACC, acetyl-CoA carboxylase; ALD, alcoholic liver disease; BAs, boswellic acids; CYP2E1, cytochrome P450 2E1; FASN, fatty acid synthase; HDL-C, high-density lipoprotein cholesterol; LDL-C, low-density lipoprotein cholesterol; TC, total cholesterol; TG, triglycerides.

Ethanol-fed mice (ALD group) revealed a significant increase in TC, TG, and LDL-C serum levels (≈ 3–4 folds; compared to control). This effect was reversed by the administration of BAs (500 mg/kg/day) which caused ≈ a 50% reduction in their levels in the ALD group. However, HDL-C serum levels decreased in the ALD group by 37%; a level which significantly increased (1.5 folds; relative to the ALD group) upon administration of BAs (500 mg/kg/day). In a similar pattern, hepatic TG is increased (≈ 3.5 folds) in the ALD group, an effect which is normalized (≈ 64% reduction) in the BAs-treated group (Fig. 2).

#### Effect of boswellic acids on the levels of adiponectin, CYP2E1, ACC-1, FASN, and miRNA-155 expression in hepatic tissue of ethanol-treated mice

Figure 2 shows an evident reduction (72%) in adiponectin hepatic concentration in the ALD group, as compared to the control group, a concentration that is significantly improved by a ≈ 2.6-fold increase in the BAs-treated group. However, the ethanol-fed mice exhibited a marked elevation (≈1.2 fold) in CYP2E1 hepatic levels relative to the control group, an elevation that noticeably fell by 32% in the BAs-treated group. Likewise, the hepatic gene expression levels of ACC-1 and FASN were ominously elevated by ≈ 5.4 and 7 folds, respectively in the ALD group, as compared to the control one, while treatment with BAs succeeded to evidently reduce them by 40 and 52%, respectively, relative to the ALD group. Also, the hepatic expression levels of miRNA-155 were significantly increased (9-fold) in the ALD group, but decreased by about 54% in the BAs-treated mice as compared to the ALD group.

#### Effect of boswellic acids on the levels of NOX1, NOX2, NOX4, phospho-p38 MAPK (Thr180/Tyr182), SREBP-1c, and PPARα in hepatic tissues of ethanol-treated mice

**Fig. 3:**
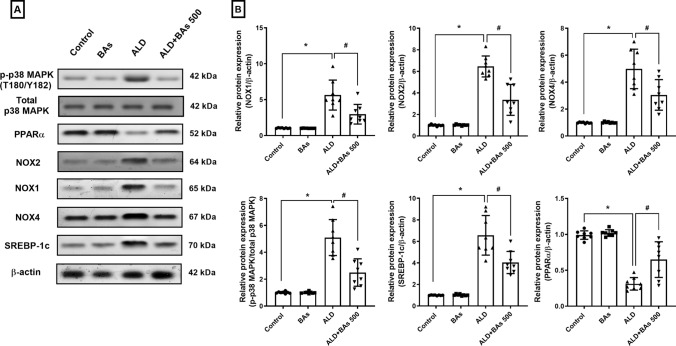
**A** Representative western blot bands and **B** quantitation of hepatic protein expression levels of NOX1, NOX2, NOX4, p38 MAPK, SREBP-1c, and PPARα following BAs treatment (500 mg/kg) in experimentally-induced ALD in mice. Data are presented as the mean ± SD (n = 8 per group; one-way ANOVA followed by Tukey’s multiple comparison test; ^*^ *p* < 0.05, vs. the control group; and ^#^ *p* < 0.05, vs. the ALD group). ALD, alcoholic liver disease; BAs, boswellic acids; MAPK, mitogen-activated protein kinase; NOX, nicotine adenine dinucleotide phosphate oxidase; PPARα, peroxisome proliferator-activated receptor alpha; SREBP-1c, sterol regulatory element-binding protein-1c.

The relative protein expression levels of NOX1, NOX2, and NOX4 were intensified by about 5–6 folds in the ALD group, as compared to the control one. These levels markedly declined in the mice that were treated with the highest dose of BAs by ≈ 40–48%, when compared to the ALD group (Fig. 3). Similar results were noticed in p-p38 MAPK (T180/Y182) and SREBP-1c relative protein hepatic content, where the ethanol-fed mice showed a marked increase in both markers (5 and 6.5-fold, respectively) when compared to the control mice, and the levels that were significantly hampered by 51 and 39%, respectively, in the BAs-treated group.

Conversely, Fig. 3 showed a marked fall in PPARα hepatic content by 68% in the ALD group, whereas the treatment with BAs restored these levels in the BAs-treated mice by about a 2-fold upturn when compared to the ethanol-treated mice.

#### Effect of boswellic acids on hepatic tissue levels of oxidative stress markers (CAT, H_2_O_2_, MDA, 4-HNE, SOD, and GPx) in ethanol-treated mice

**Fig. 4:**
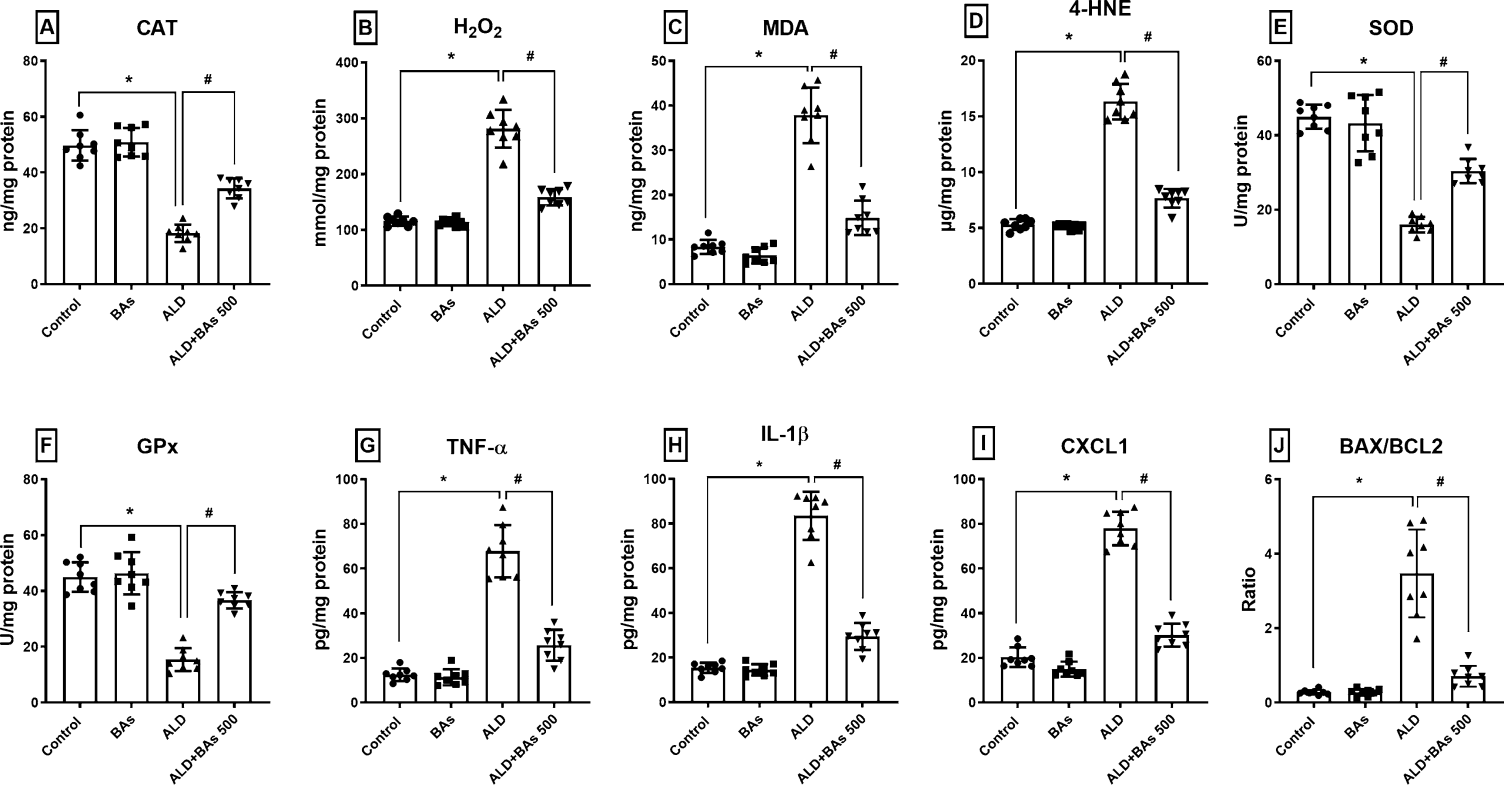
Effect of BAs (500 mg/kg) on the hepatic levels of **A** CAT, **B** H_2_O_2_, **C** MDA, **D** 4-HNE, **E** SOD, **F** GPx, **G** TNF-α, **H** IL-1β, **I** CXCL1, and **J** BAX/BCL2 in experimentally-induced ALD in mice. Data are presented as the mean ± SD (n = 8 per group; one-way ANOVA followed by Tukey’s multiple comparison test; ^*^p < 0.05, vs. the control group; and ^#^*p* < 0.05, vs. the ALD group). ALD, alcoholic liver disease; BAs, boswellic acids; BAX, BCL2-associated X protein; BCL2, B-cell lymphoma 2; CAT, catalase; CXCL1, C-X-C motif chemokine ligand 1; GPx, glutathione peroxidase; 4-HNE, 4- hydroxynonenal; H_2_O_2_, hydrogen peroxide; IL-1β, interleukin 1 beta; MDA, malondialdehyde; SOD, superoxide dismutase; TNF-α, tumor necrosis factor-alpha. *p* < 0.05 Signficance levels are superscripted

Figure 4 showed that the tissue levels of CAT, SOD, and GPx were noticeably diminished in the ethanol-fed mice by ≈ 63–66%. In contrast, H_2_O_2_, MDA, and 4-HNE tissue levels were raised by ≈ 2.4, 4.5, and 3.1 folds, respectively, when compared to the control group. These effects were corrected by the administration of BAs (500 mg/kg/day) which produced ≈ a 2-fold rise in CAT, SOD, and GPx levels, while causing ≈ 43.6, 61, and 53% reduction in H_2_O_2_, MDA, and 4-HNE levels, respectively, relative to the ALD group.

#### Effect of boswellic acids on hepatic tissue levels of TNF-α, IL-1β, CXCL1, and BAX/BCL2 ratio in ethanol-treated mice

As shown in Fig. 4, the ALD group presented a distinct increase in hepatic tissue levels of TNF-α, IL-1β, CXCL1, and BAX/BCL2 ratio by 5.4, 5.4, 3.8, and 12.5 folds, respectively, relative to the control mice. However, the levels of the BAs-treated mice (500 mg/kg/day) were markedly lessened their levels by ≈ 62, 65, 61, and 80%, correspondingly, when compared to their concentrations in the ALD group.

#### Effect of boswellic acids on phospho‑nuclear factor kappa B p65 (Ser276) and cleaved caspase-3 immunoexpression

**Fig. 5:**
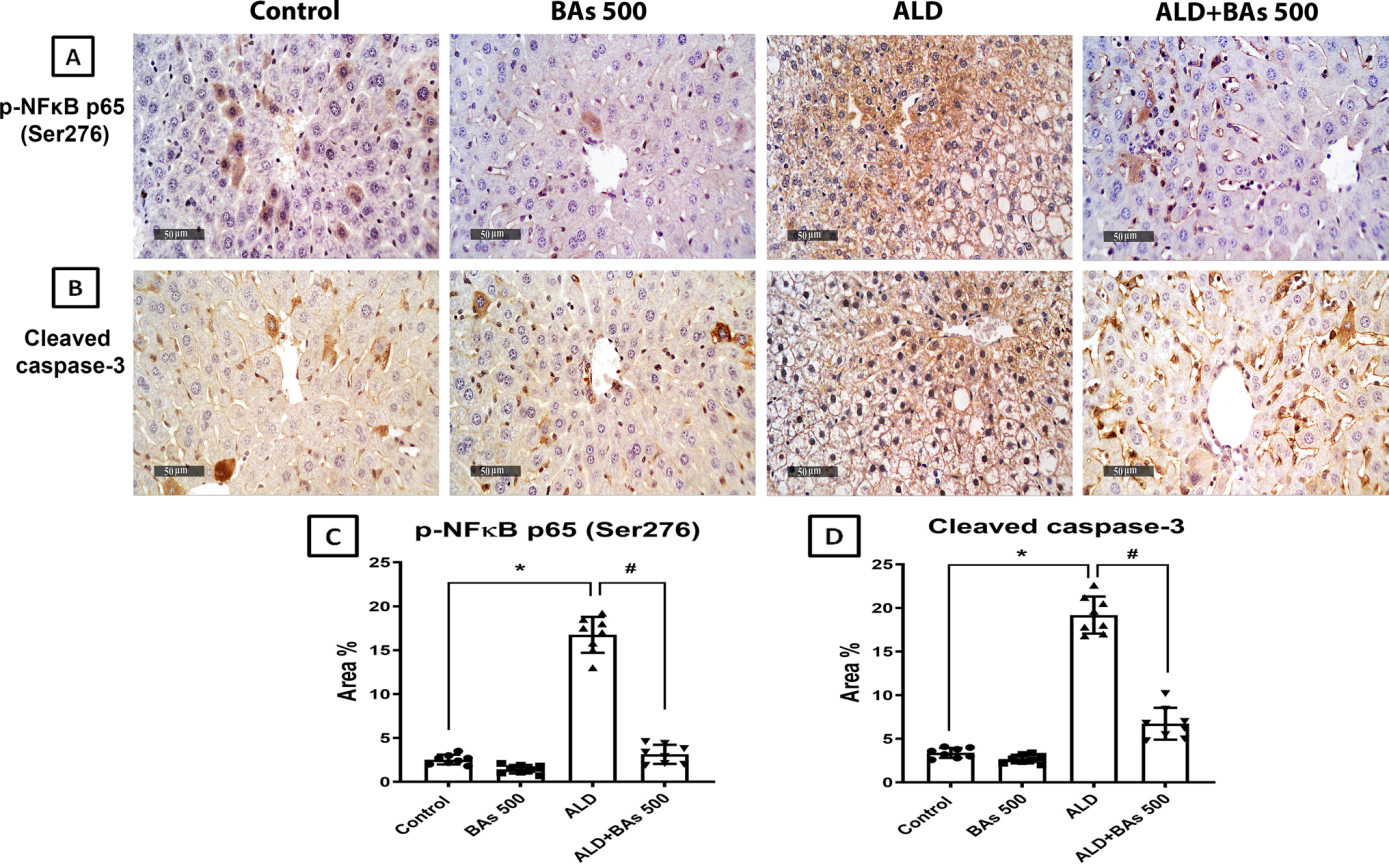
Photomicrographs of immunohistochemical staining (**A**, **B**; X400) and the area percentage of immunoexpression levels of hepatic **C** p-NFκB p65 (Ser276) and **D** cleaved caspase-3, following BAs treatment (500 mg/kg) in experimentally-induced ALD in mice. Data are presented as the mean ± SD (n = 8 per group; one-way ANOVA followed by Tukey’s multiple comparison test; ^*^p < 0.05, vs. the control group; ^#^*p* < 0.05, vs. the ALD group). ALD, alcoholic liver disease; BAs, boswellic acids; p-NFκB, phosphorylated nuclear factor kappa B. *p* < 0.05 Signficance levels are superscripted.

The photomicrographs and immunoreactivities of both p-NFκB p65 (Ser276) and cleaved caspase-3 were assessed and demonstrated in Fig. 5. The ALD group showed a significant increase in the immunoreactivities (area %) of p-NFκB p65 (Ser276) and cleaved caspase-3 by ≈ 6.6 and 5.5 folds, respectively, as compared to the control group. However, these effects considerably declined by ≈ 81% and 65%, respectively, upon administration of BAs (500 mg/kg/day), relative to the ALD group.

## Discussion

Halting the impact of alcohol at the stage of steatosis can prevent or slow down the progression of ALD to a more severe irreversible state. Thus, the introduction of novel prophylactic agents that can target the molecular pathways implicated at this stage can be of immense value. In our study, we introduced the novel protective impact of BAs against experimentally-induced ALD in mice by investigating a dose-response effect of three BAs doses (125, 250, and 500 mg/kg/day). The highest dose was the most hepatoprotective and hence was further investigated to understand its molecular mechanism of action.

Importantly, we revealed for the first time that the protective effect of BAs may be related to halting NOX/p38 MAPK/miR-155 and ACC-1/FASN signaling axes, and correcting PPARα expression which was reflected in reduced oxidative stress, inflammation, and apoptotic indices. Noteworthy, our new findings of these hepatoprotective actions of BAs in an experimentally-induced ALD added to other research on its preventative effects in other liver diseases. For example, earlier studies stated that BAs ameliorated multiple agents-induced hepatotoxicity models via reducing the expression of hepatic transforming growth factor beta, phosphorylated c-Jun N-terminal kinase, TNF-α, NFκB, and IL-6, while enhancing the nuclear factor E2-related factor 2/heme oxygenase 1 axis (Chen et al. 2017; Kumar et al. 2017; Barakat et al. 2018; Eltahir et al. 2020). Meanwhile, in a non-alcoholic fatty liver experimental model, BAs restored the reduced glutathione, and diminished MDA levels and other inflammatory mediators as TNF-α, IL-6, cyclooxygenase 2, and inducible nitric oxide synthase in hepatic tissues (Zaitone et al. 2015).

Alcohol can induce either weight gain or loss. Alcohol-induced weight loss can be attributed to the continued elevated levels of BAC and appetite suppression, as previously reported following daily injection of ethanol in rats (Nelson et al. 2016). This was augmented by our results, in which a significant increase in BAC together with weight loss of ethanol-fed mice was observed. The current model induces liver injury and steatosis (Cabre et al. 2021), which was observed in the increased liver index in the ethanol-fed group. This came in agreement with previous studies in which chronic plus binge ethanol administration showed a significantly higher liver index (Xu et al. 2018). The high dose of BAs showed a significantly reduced liver index when compared to both the ethanol-fed mice and the low-dose treated group. BAs effect on body weight did not differ significantly from the control group, however, the administration of BAs high dose to the ethanol-fed mice showed significant improvement in body weight loss, when compared to the ethanol-fed group.

Ethanol is mainly metabolized by ADH into acetaldehyde, and ALDH catalyzes the transformation of acetaldehyde into acetate, which can then be released into the circulation affecting vital organs. In our study, the ethanol-fed group showed reduced hepatic ADH and ALDH activity. This can be attributed to the theory that the chronic ethanol intake can inhibit the activity of these enzymes (Lee et al. 2013). Notably, the treatment with BAs managed to reverse these effects in a dose-dependent manner, showing the highest activity of both enzymes with BAs high dose. Such results can justify the observed dose-dependent decline in the BAC in the treated groups.

The observed increase in the serum levels of liver function enzymes of the ethanol-fed mice is one of the common features of alcoholic hepatitis (Giannini et al. 2005). In this study, the present hepatocellular degeneration and inflammatory cells infiltrate further augments the presence of hepatic injury in the ALD group. Inversely, prior administration of BAs protected the hepatocytes against ethanol-induced pathological damage in a dose-dependent manner. The hepatoprotective impact of BAs was demonstrated by a substantial decline in raised liver enzymes, and the restoration of normal hepatocyte histological features. Our findings support prior studies that showed BAs to be hepatoprotective against a variety of hepatotoxic agents (Abdel-Daim et al. 2018; Eltahir et al. 2020).

The primary response of the liver to alcohol abuse is steatosis or fat accumulation, principally TG. This occurs via elevating the NADH/NAD^+^ ratio which interrupts the β-oxidation of fatty acids in the liver and sensitization of *de novo* lipogenesis from non-lipid precursors (You and Arteel 2019). This dyslipidemic state was previously reported (Zhou et al. 2021) and is evident in the current study in the elevated lipid profile markers, mainly TG, and the hepatocellular steatosis observed in the histopathological picture. Herein, the high dose of BAs offered potent hypolipidemic effects against ethanol by significantly improving the previously altered lipid profile and preserving the histological architecture. Recently, several research efforts have proved the *Boswellia* species’ hypolipidemic capability. According to animal research, the aqueous extract of *B. serrata* had a substantial hypocholesterolemic effect as well as a positive impact on serum HDL-C (Pandey et al. 2005; Mohamed et al. 2021). Clinically, when diabetic individuals were treated with *B. serrata* extract, their lipid profile was held near standard norms, resulting in a large increase in HDL levels and a significant decrease in TC and LDL-C (Ahangarpour et al. 2014).

CYP2E1 is highly expressed in the liver and contributes significantly to the pathogenesis of ALD via excessive production of free radicals (Harjumäki et al. 2021). This was evident in our study and prior ones (Sun et al. 2017; Nagappan et al. 2018; Shi et al. 2022), where enhanced CYP2E1 expression was associated with the depletion of GPx, SOD, and CAT, and increased levels of H_2_O_2_, MDA and 4-HNE in ethanol-fed mice. Throughout this study, the high dose of BAs effectively diminished the upregulated CYP2E1 in the ethanol-fed mice and restored the antioxidant defense mechanism as well. Our findings back up previous research that indicates the antioxidant capacity of BAs in different body organs (Chen et al. 2016; Wei et al. 2020), while another study linked this antioxidant effect of BAs to its ability to normalize CYP2E1 levels, hence relieving hepatocellular damage caused by other agents (Chen et al. 2017).

The ethanol-fed mice showed increased protein expression levels of NOX1, 2, and 4 in the liver. Enhanced NOX1 and 4 expression is correlated to heightened mitochondrial ROS production, whereas NOX2 expression is enhanced in response to remarkable neutrophil infiltration in the liver following binge ethanol administration, and hence mediates an oxidative surge (Yang et al. 2022). Such effects were previously highlighted in response to ethanol intake (Wang et al. 2017, 2021a). To the best of the authors’ knowledge, this is the first work to show that BAs inhibits NOX1 and 4 protein expression, confirming our current findings that highlight the potent antioxidant properties of BAs. Furthermore, the newly discovered inhibitory impact of BAs on NOX2 might augment its anti-inflammatory properties.

The molecular mechanisms involved in the alcohol-induced fatty liver include crosstalk between the upregulation of SREBP-1c, ACC-1, and FASN and the downregulation of PPARα (Ji et al. 2006; Meng et al. 2020; Wang et al. 2021c), which was evident in our reported data in the ALD group. Ethanol-induced CYP2E1 overexpression and the subsequent oxidative stress can be responsible for the decreased levels of adiponectin (Tang et al. 2012), which was observed in the ALD group in our study. The reduced protein expression of PPARα together with the enhanced activity of SREBP-1c can be attributed to the increased expression of CYP2E1 and the halted expression and secretion of adiponectin (Liangpunsakul 2015; Gamberi et al. 2018; Meng et al. 2020), which was reported both in our study and in agreement with previous works (Lee and Lee 2015). The benefit of *B. serrata* extract in avoiding obesity, hyperlipidemia, and steatosis may be mediated by increasing adiponectin levels (Gomaa et al. 2019). Such results corroborated our outcomes that BAs restored the adiponectin levels, elucidated by mitigating the ethanol effect on both PPARα and SREBP-1c activity, which coincided with our noticed inhibitory effect of BAs on ethanol-induced CYP2E1 upregulation and oxidative stress. In contrast to the ethanol effect on the lipogenesis genes, BAs treatment downregulated ACC-1 and FASN gene expression. In our investigation, the enhancing effect of BAs on PPARα agreed with an earlier report (Thabet et al. 2020), but its suppressive effect on SREBP-1c levels as well as ACC-1 and FASN expression was a breakthrough discovery for us. Altogether, our data support the BAs’ key role in hindering ethanol-induced steatohepatitis by hitting several molecular targets.

The noticed reduction of PPARα expression in the ALD group in our study can be attributed to the elevated expression of miR-155, as previously reported (Bala et al. 2016). Indeed, miR-155 levels in the blood have been observed to be higher in healthy people after binge drinking and in animal models of liver injury (Bala et al. 2012). Furthermore, previous studies showed that it is one of the most implicated miRNAs in the pathogenesis of ALD (Mandrekar and Szabo 2009; Szabo and Bala 2013). Fortunately, we discovered that treatment with 500 mg of BAs inhibited the increased levels of miR-155, most likely via restoring PPARα function, revealing that BAs can mitigate the damaging consequences of miR-155 in ALD pathogenesis.

The imbalanced redox status in ALD is intertwined with enhanced activity of NFκB and p38 MAPK, as well as reduced activity of PPARα; thus, aggravating hepatic inflammation (Ambade and Mandrekar 2012). The inhibitory effect of ethanol on PPARα halts its suppressing effect on NFκB p65 (Nakajima et al. 2004). Alcohol intake is known to activate KCs; an innate immune response, which orchestrates hepatic inflammation via enhancing the translocation of the gut endotoxin, lipopolysaccharide, to the liver and its subsequent binding to toll-like receptor 4, leading to enhanced NFκB activity and the downstream increase in TNF-α (Zeng et al. 2016). Furthermore, the increased activity of NFκB and TNF-α levels activate HSCs, which leads to increased levels of CXCL1, and subsequent neutrophil infiltration (Gao and Bataller 2011). This inflammatory status is revealed in our study and previous reports through the elevation of TNF-α and IL-1β (Abdelhamid et al. 2021), and the chemokine CXCL1 (Sangineto et al. 2020) in the ethanol-fed mice. Also, ethanol-induced upregulation of miR-155 expression in macrophages is linked to the increased TNF-α release, probably via activating KCs as previously reported (Bala et al. 2011). MiR-155 is the main regulator of KCs activation and function, as it inhibits the expression of multiple NFκB inhibitory regulators, allowing NFκB induction (Mahesh and Biswas 2019).

In this study, the restored antioxidant defense mechanism by the treatment with 500 mg of BAs halted the hepatic inflammatory status in ethanol-fed mice. BAs’ anti-inflammatory activity was reflected in the suppression of NFκB p65 and p38 MAPK, thereby hindering the TNF-α signaling downstream. Additionally, treatment with BAs reduced the production of the chemokine CXCL1; the major chemoattractant for neutrophils (Sawant et al. 2016), and IL-1β, supporting its potent anti-inflammatory impact. BAs were reported to have anti-inflammatory properties in the brain, heart, and liver by inhibiting NFκB and the downstream cascade (Chen et al. 2017; Wei et al. 2020; Siddiqui et al. 2021), while another study found that the whole extract of *B. serrata* impedes the levels of TNF-α, IL-1β, and MAP kinases in human peripheral blood mononuclear cells and mouse macrophages (Gayathri et al. 2007). However, we detected a unique anti-inflammatory effect of BAs on the chemokine CXCL1 in our study and thus, can affect neutrophil infiltration.

Collectively, this perturbed redox status and hepatic inflammation led to hepatotoxicity via increased release of cytochrome C and activation of the apoptotic signaling cascade (Namachivayam and Valsala Gopalakrishnan 2021). This was supported by the reported increase in BAX to BCL2 ratio and caspase-3 activity in ethanol-fed mice both in our study and previous works (Wu et al. 2019). On the contrary, BAs exerted a notable anti-apoptotic activity in ethanol-fed mice, evidenced by restriction of caspase-3 activity and BAX/BCL2 ratio, which comes in agreement with recent research on BAs’ antiapoptotic properties in various injury models (Ahmed et al. 2020; Rajabian et al. 2020).

In conclusion, the use of BAs, derived from *B. serrata*-standardized extract, has garnered considerable attention in the treatment and prevention of various chronic diseases. In our study, BAs provided remarkable hepatoprotective actions against ethanol liquid diet-induced ALD, in a dose-dependent manner. Upon revealing its underlying mechanism, the highest dose of BAs was observed to interfere with the regulation of NOX/p38 MAPK/ACC-1/FASN pathways and miR-155 expression along with restoration of PPARα-mediated hepatic antioxidant activity and lipid metabolism. Additionally, the impressive anti-inflammatory and anti-apoptotic properties exerted by BAs might advocate its defensive mechanism against ethanol-mediated hepatic injuries. We can, therefore, anticipate that BAs would exert a beneficial impact on ALD via its dominant hepatoprotective action, in which additional investigations are warranted to corroborate the usefulness of BAs in patients suffering from chronic liver diseases.

## Supplementary Information

Below is the link to the electronic supplementary material.

Supplementary material 1 (PDF 812.1 kb)
