# Supplementary figures and images for: Regulation of NOX/p38 MAPK/PPARα pathways and miR-155 expression by boswellic acids reduces hepatic injury in experimentally-induced alcoholic liver disease mouse model: novel mechanistic insight
Source: Arch Pharm Res. 2023 Mar 23;46(4):323–38. doi: 10.1007/s12272-023-01441-6 (PMC10123034; doi:10.1007/s12272-023-01441-6)

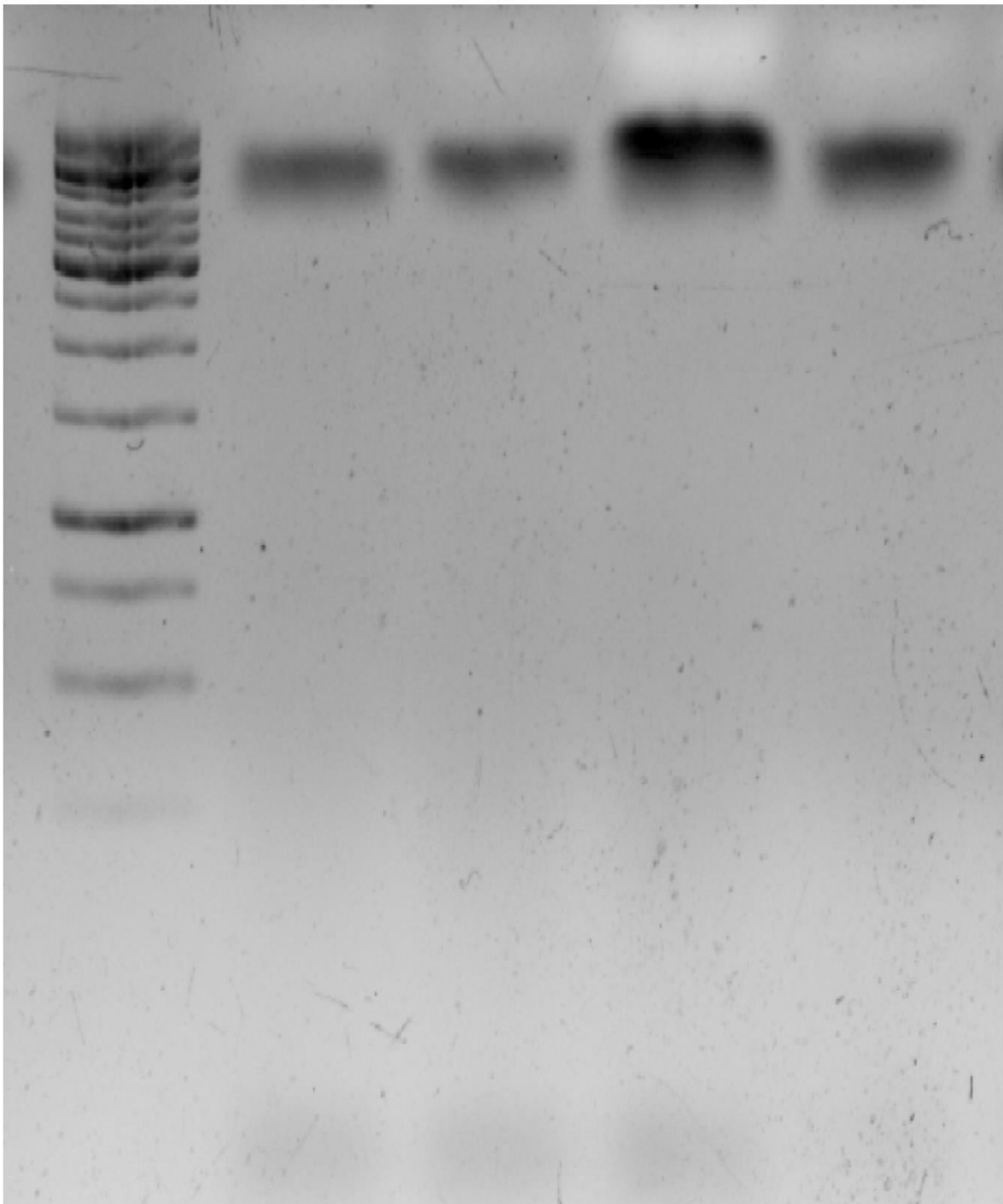

**SREBP-1c**

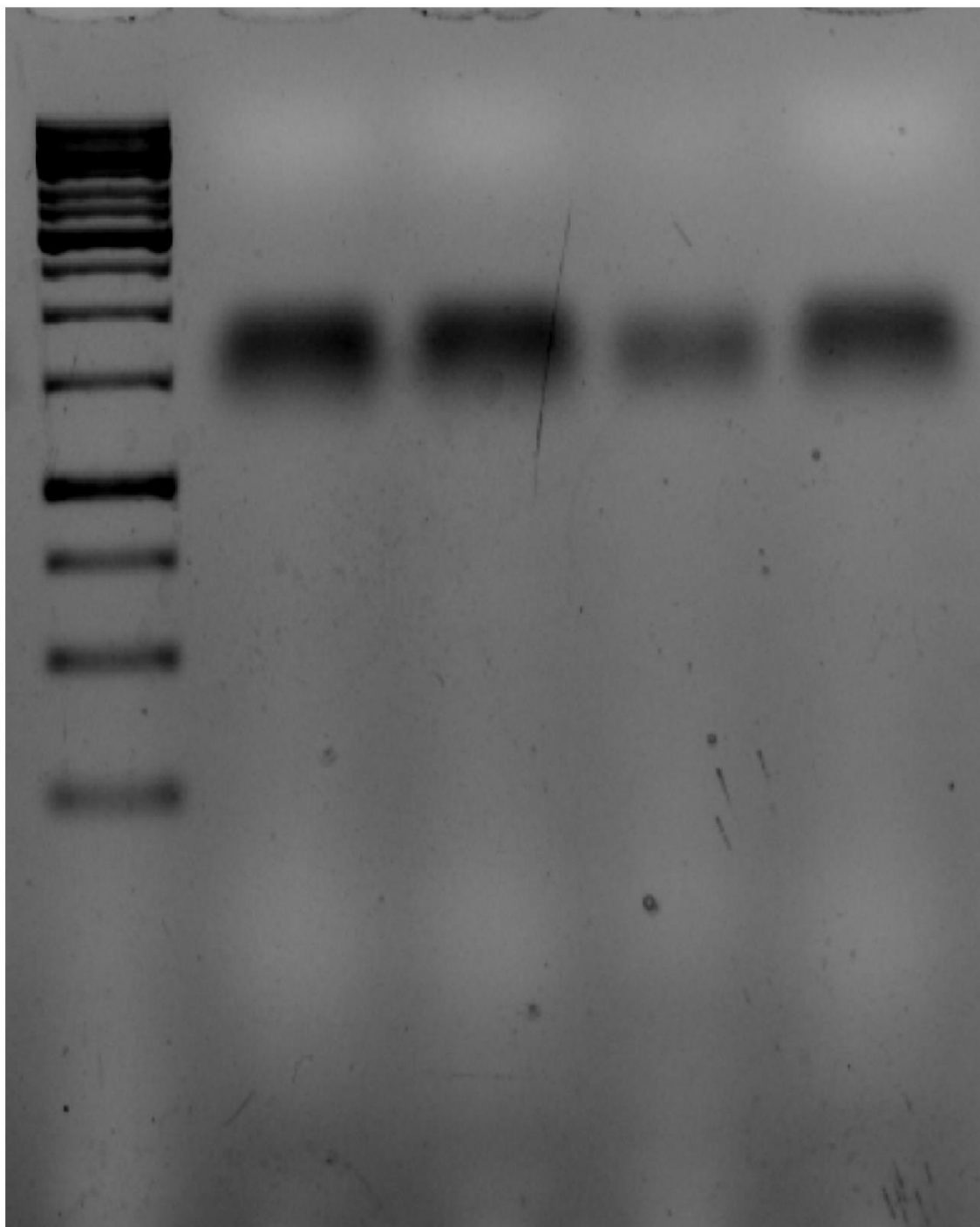

PPAR alpha

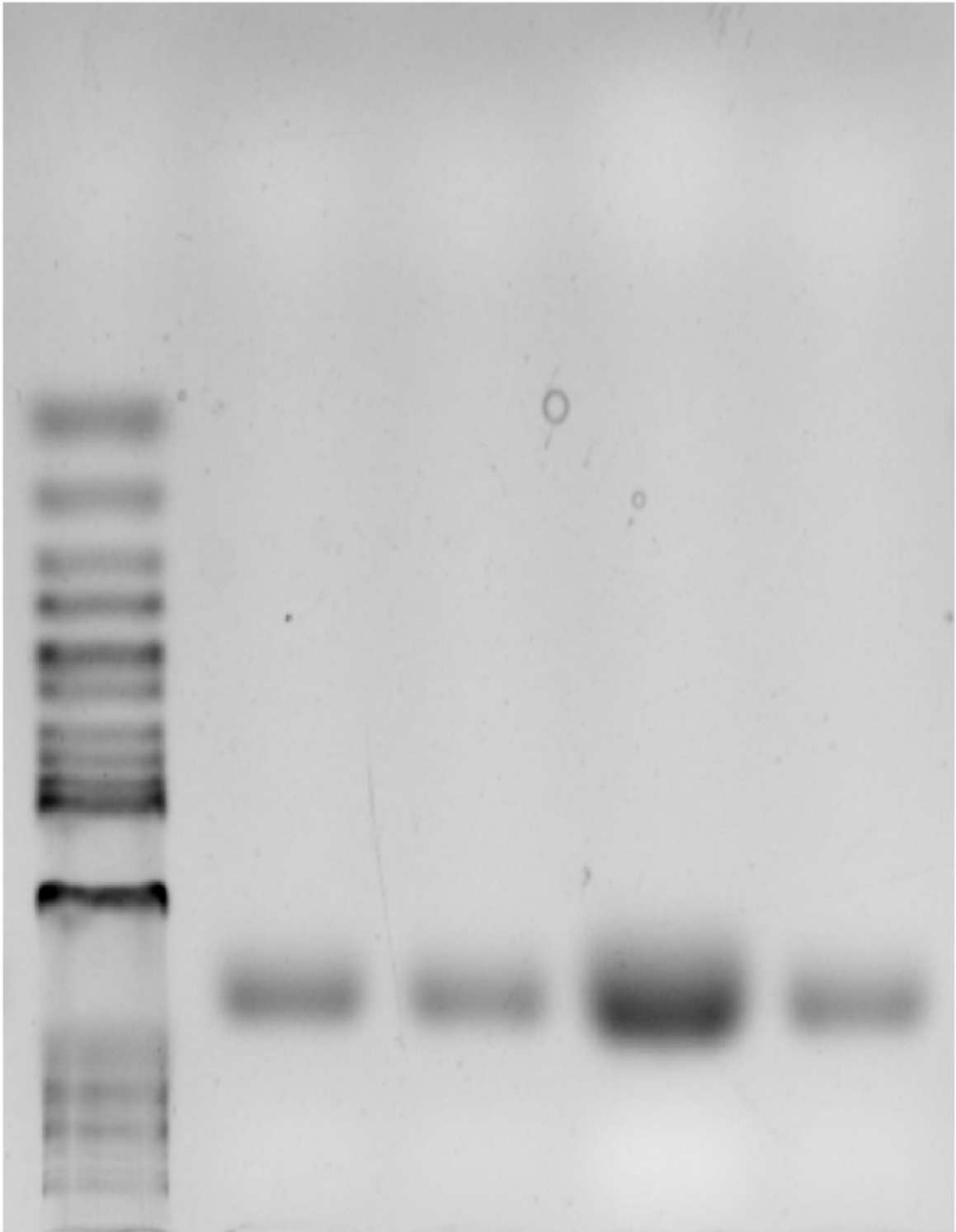

**Phospho p38 MAPK**

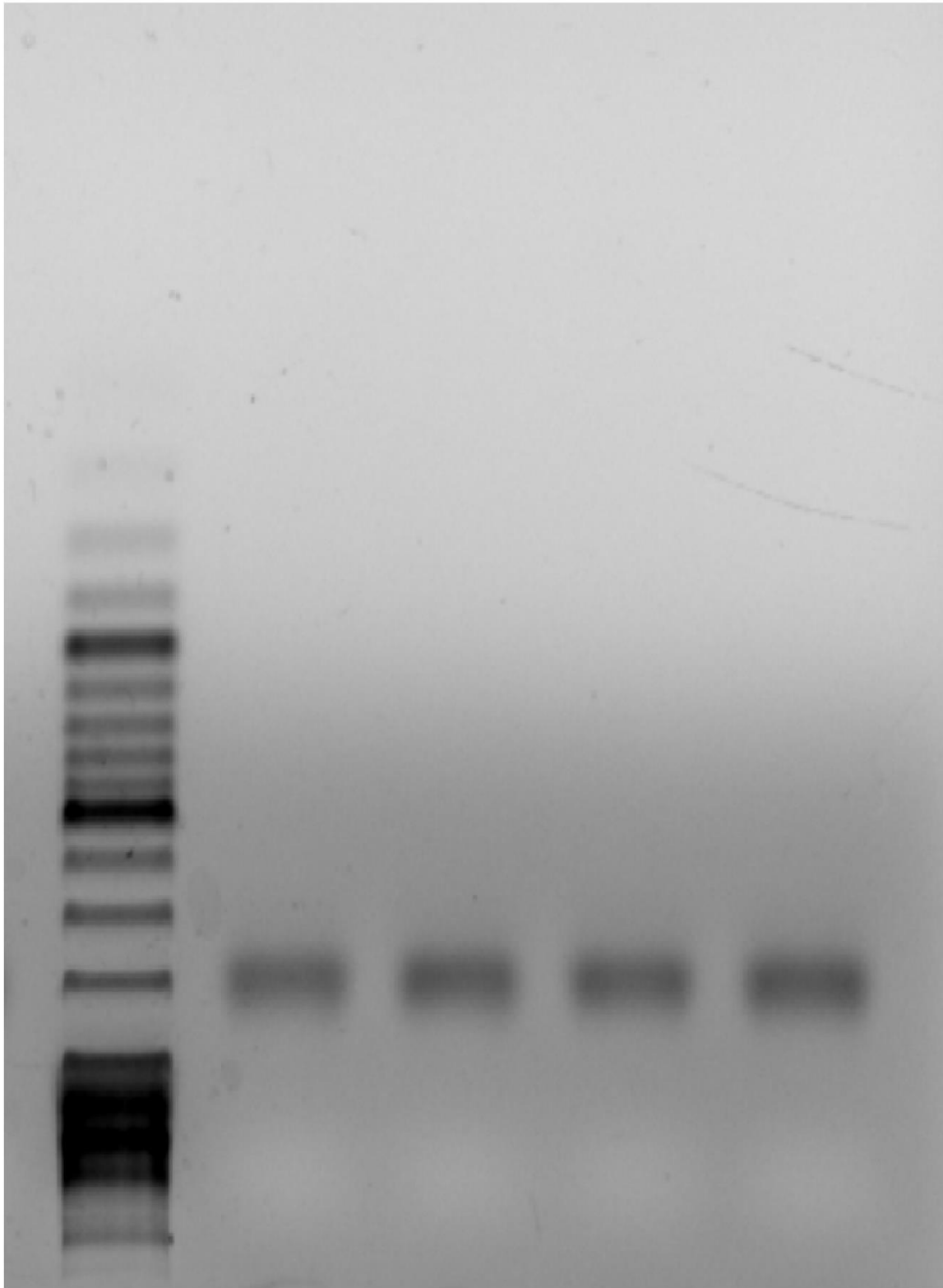

**Total p38 MAPK**

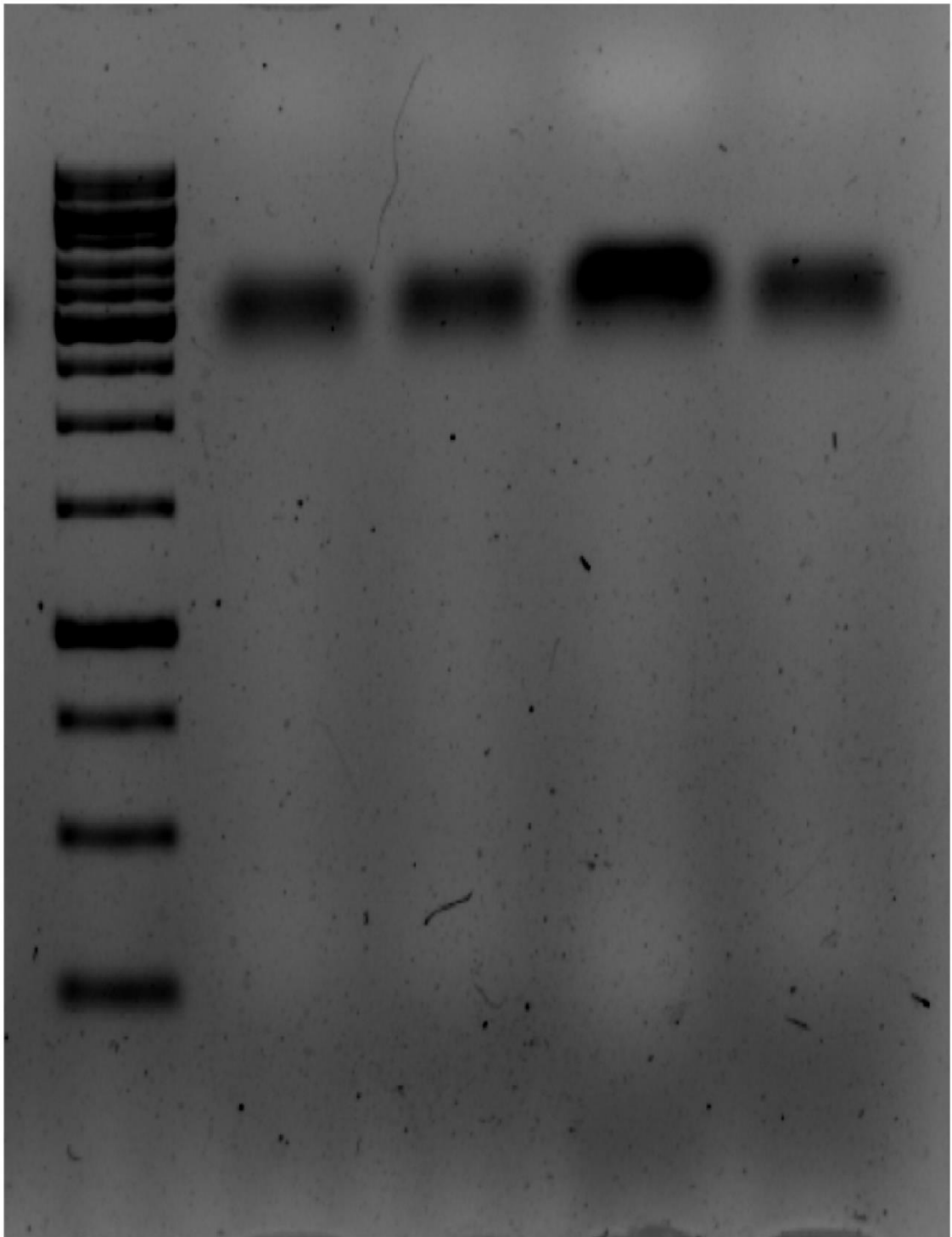

**NOX4**

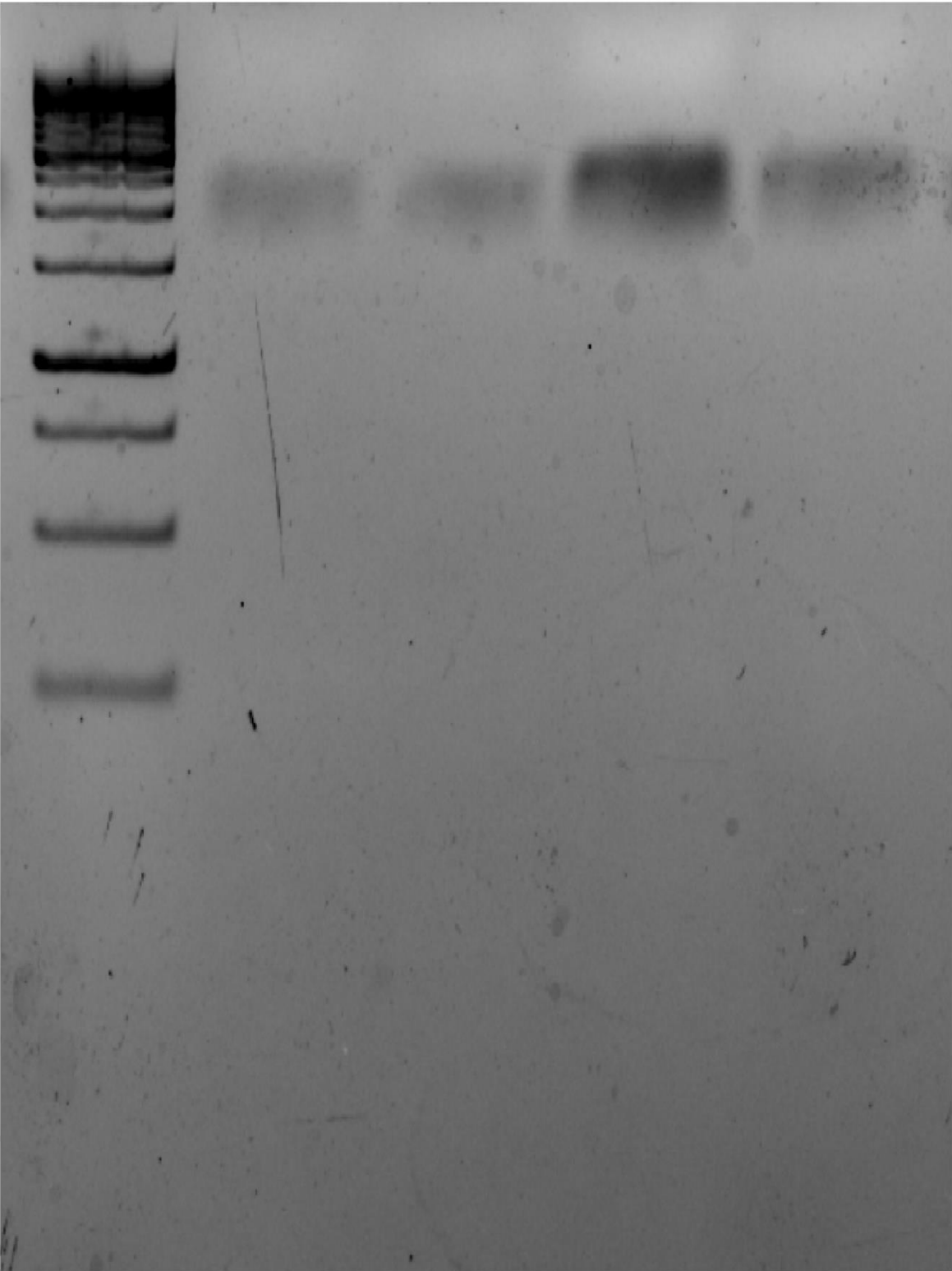

**NOX2**

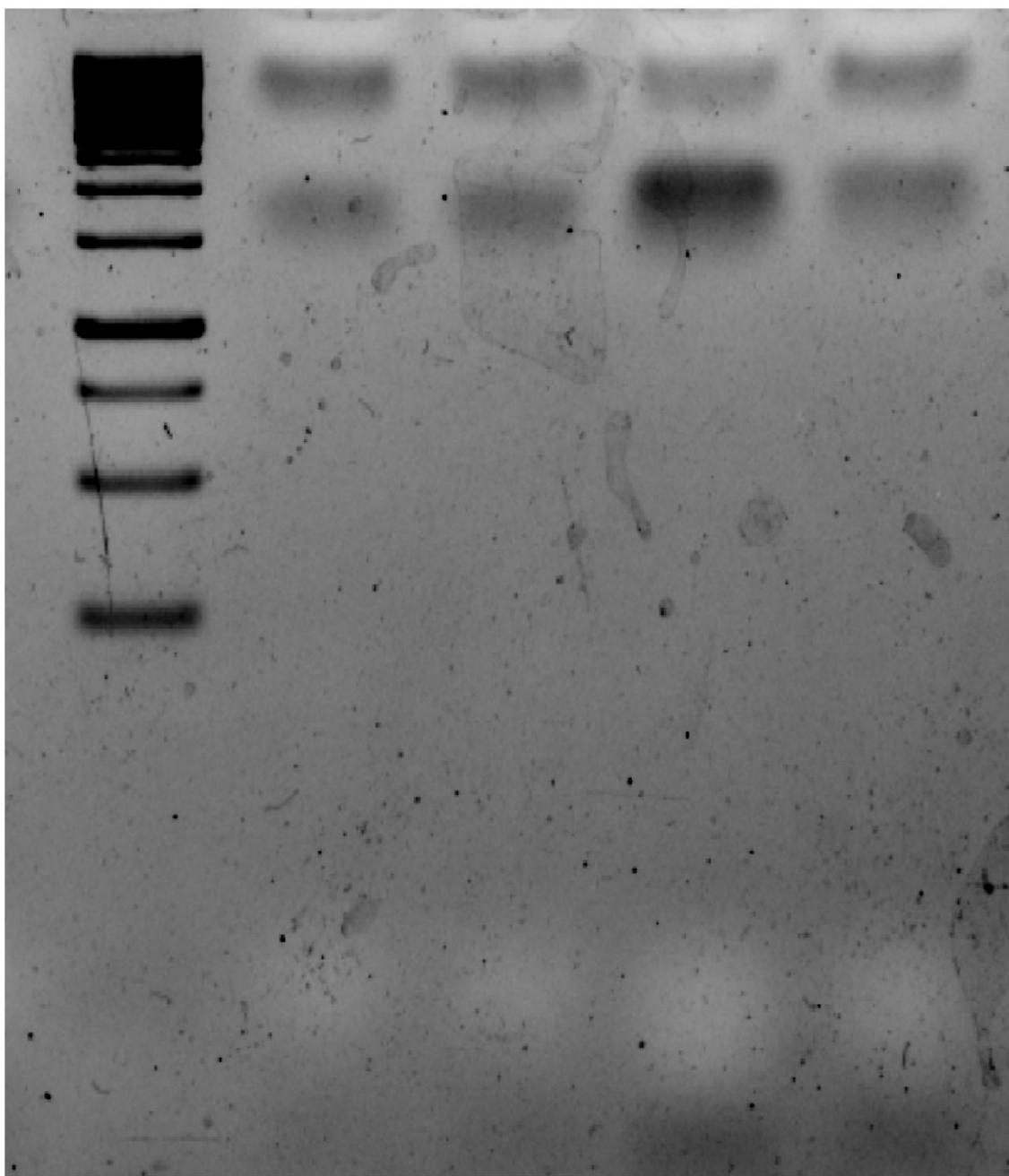

**NOX1**

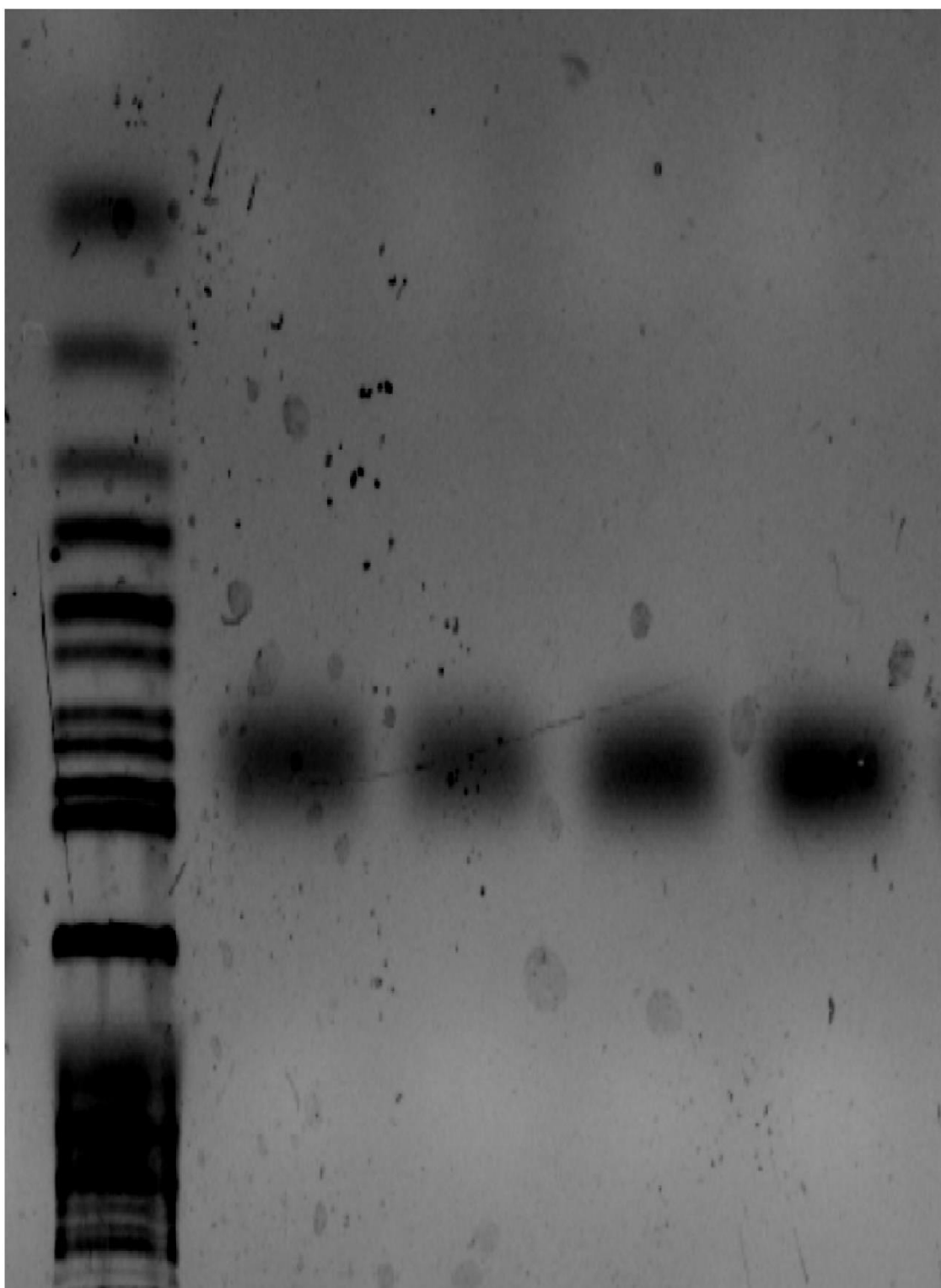

**Beta actin**

Supplement: Supplementary file 1 — Supplementary material 1 (PDF 812.1 kb) [file 12272_2023_1441_MOESM1_ESM.pdf]
